# Is Post-Burn Scarring a Research Priority?

**DOI:** 10.3390/ebj3020030

**Published:** 2022-05-03

**Authors:** Amber E. Young, Robert M. T. Staruch

**Affiliations:** 1 Centre for Surgical Research, Population Health Sciences, Bristol Medical School, Bristol BS8 1UD, UK; 2 Bristol Royal Hospital for Children, Bristol BS8 1QU, UK; 3 Department of Engineering Science, University of Oxford, Oxford OX3 7LD, UK; robert.staruch@eng.ox.ac.uk; 4 Nuffield Department of Orthopaedic, Musculoskeletal & Rheumatological Sciences (NDORMS), University of Oxford, Oxford OX3 7LD, UK

## 1. Introduction

National and international research budgets are insufficient to approve all requests for funding, even if a methodology is of high quality and the outputs are likely to have an impact on improving patient outcomes [1]. Funding organisations must therefore make decisions about the best way to prioritise competing research needs and to ensure that scarce healthcare research funding is focused on areas relevant to patients and known to lack evidence. Agreeing on priority areas for healthcare research funding is key to reducing research waste and improving impact [2]. If research addresses questions of relevance to patients and clinicians, decision-makers will be better equipped to design and deliver health services that meet their needs. Governments and funders are more likely to listen to the results of a large-scale project to achieve consensus on research needs undertaken with patients and healthcare staff [3].

A priority setting exercise has not yet been undertaken for burn care research. A consequence of this is that burn care studies are often small; are conducted in a single centre; and focus on areas of clinician interest, without evidence for patient relevance [4,5]. If a research question can be shown to answer a highly ranked priority area that has been shown to lack evidence and to align with patient and clinician views, funding can be diverted to large multi-centre collaborative studies [6]. Another benefit of research prioritisation if undertaken internationally is the potential for harmonisation of clinical research at the global level [7].

The aim of this position paper is to explore methods for assessing the areas on which to focus research within burn care and how issues related to post-burn scarring can be assessed in terms of patient importance and the need for national or international research funding.

## 2. The Priority-Setting Processes

The methodology for prioritising healthcare research is still evolving. Strategies generally consist of survey-based collection of candidate priorities, a method for ranking of these priorities in terms of stakeholder importance and a final meeting to achieve consensus on the top ranked research priorities [8]. The priorities set will be used with governments and research funders to set an agenda for research to be undertaken in the healthcare area explored. There have been differences shown in response to the request for potential research topics between patients and clinicians; in a review of JLA PSPs, the authors found that patients focused more on symptoms and function than on disease, while clinicians focused on disease management [9]. Most methodologies therefore support the input of patient views as well as those from clinicians [10,11]. There is more heterogeneity with respect to the involvement of international participants [12,13,14,15,16]. Decisions about the scope of priority setting exercises in terms of international participation are commonly related to the nature of the disease, resource availability and views of the organising committee.

**Figure 1:**
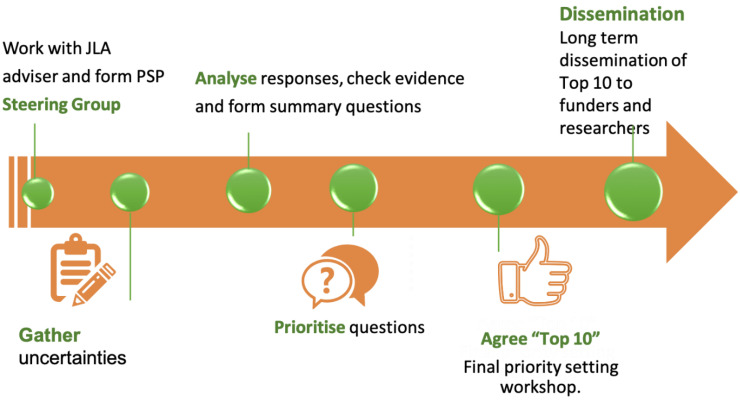
Phases of a JLA PSP.

The James Lind Alliance ((JLA) https://www.jla.nihr.ac.uk/, accessed on 1 February 2022), launched in 2004, is a UK healthcare priority setting partnership often working internationally and supported by the National Institute for Health Research (NIHR). It encourages collaboration between patients and healthcare workers in priority setting partnerships (PSP) to identify research priorities in clinical areas that lack evidence [3,9,17,18]. The process includes development of a steering group, agreement on the project scope, gathering clinical uncertainties from stakeholders, interim priority setting results and a final priority setting consensus meeting (Figure 1). Importantly, this final meeting is led by the JLA and only involves the steering group if prior agreement is obtained from stakeholders. This minimises the steering group introducing bias in the priority setting process. The final top ten priority themes are not worded as questions but instead are healthcare areas that represent true evidence gaps ranked as most important by all stakeholders. The final priorities are them disseminated to stakeholders and international funders. More than 100 priority setting partnerships (PSPs) have so far been established through the JLA to identify, verify and prioritise questions in different clinical fields [19].

Other methods for setting the research priority exist. Examples include the Child Health and Nutrition Research Initiative (CHNRI); the Council on Health Research for Development (COHRED) [20]; the VU University, which provides the ‘Dialogue Model’ framework [21]; the Essential National Health research (ENHR) approach; and the 3D combined approach matrix (CAM) [7]. The CHNRI method differs from other research prioritisation methods through the introduction of a set of criteria to discriminate between competing research options [12,22,23]. The five criteria are (i) answerability, (ii) effectiveness, (iii) deliverability, (iv) the potential for a substantial reduction in disease burden and (v) the impact on equity. There are also different methods used for reaching consensus. The JLA uses the more common Delphi method, which uses stakeholder feedback in iterative surveys to achieve agreement on items [24]. Other authors have used the Hanlon Process of Prioritisation (HPP) [25]. The HPP formally weighs prevalence, seriousness and feasibility of a given research question. The EHNR process, aimed at setting national research agendas, follows an iterative process of *‘systematic and scientific assessment of health status, health systems and health research systems together with systematic and scientific analysis of use demands, felt needs and values [20,26]’.* The ENHR methodology forms part of the CAM approach. The 3D cam combines three pillars: the process of setting priorities, the tools for setting priorities and the context of these priorities. The process combines these into a matrix that takes into account the public health, institutional and equity perspectives to inform about setting priorities [27].

## 3. Priority Setting in Burn Care—What Are the Advantages?

Research prioritisation is a fundamental part of the World Health Organization’s goals for achieving equity in healthcare. Through equity in research, we can achieve equity in healthcare.

Generally, the lists created from research prioritisation consist of broad questions that allow for a variety of experimental approaches toward seeking for answers or consensus. This alleviates concerns that such prioritisation exercises box out specific fields. Instead, it gives ample room for researchers to reframe their work in the context of these prioritise. Alternatively, it allows groups to plan and initiate novel clinical trials to directly answer these research areas. In short, it provides clarity and direction for conducting large multi-centre clinical trials.

More subtly, research prioritisation narrows cultural, class and racial bias in research. Classical mechanisms for allocating research funding—through funding grants or scientific advisory panels—concentrate bias amongst a narrow group of individuals. Historically, these may have been male-dominated from a particular social class. Research-prioritisation methodologies reduce this bias by engaging with broader experiences—both from clinical and patient perspectives. Such efforts hopefully narrow health inequalities by pushing us to reduce demographic blind spots and to ensure that a variety of voices and perspectives are heard. This is a considerable advantage to research prioritisation.

Challenges exist in agreeing upon priorities in global burn care. Many common interventions lack synthesised evidence and could all be candidate research priorities. The scope of the priority setting exercise could potentially involve all injury causations, prevention, first aid as well as secondary and tertiary care. This generous scope may make a research prioritisation exercise too unwieldy to be practical. The burden of burn injuries is in lower-income countries, and there should be a reasonable expectation that participants from these countries should be included in a research setting agenda exercise. Access to clinicians and patients in these countries is complex. To answer the most important questions in global burn care, recruitment and involvement in lower income countries is essential. All countries should prioritise involvement in research work that impact patients globally. Nevertheless, the nuances of healthcare disparities between MEDCs and LEDCs may subtly influence how individual nations prioritise research funding (such as funding local prevention over cure) [28,29].

One hurdle encountered by multi-centre clinical trials is that of achieving recruitment. As burn care impacts patients globally, the potential pool of patients that could be recruited to answer key clinical questions is perhaps exponentially larger. As such, global research prioritisation engages partner nations early for clinical collaborations and potential recruitment networks. As lower-income countries should engage in answering these prioritised research questions, the top 10 list aids countries in distilling the use of their research budget. As an example, governments could focus their efforts on funding one particular multi-centre clinical trial prior to engaging the next—this could free up funding to support local preventative interventions. Hence, the benefits are two-fold, both in economic and strategic prioritisation.

A global priority setting process following the James Lind Association methodology and funded by the NIHR is starting in the UK now. The protocol can be accessed through the JLA website (https://www.jla.nihr.ac.uk/priority-setting-partnerships/burn-injury-global/, accessed on 1 February 2022). Topics chosen for importance and lack of evidence in such a PSP might include acute management fluid resuscitation [30], diagnosis and management of wound infection [31], nutrition [32], wound management [33], psychosocial well-being [34] and quality of life [35].

## 4. What Are the Drawbacks of Research Prioritisation in Burn Care

There are criticisms levelled at research prioritisation exercises. First, although they attempt to engage patients and carers, there are no mandatory minimums set for patient or carer recruitment to the process. As such the degree to which these groups influence the final top 10 can vary between PSPs. This drawback also applies to the clinical stakeholders and responders. The inherent bias of clinical stakeholders may skew their responses and how they prioritise questions. The recruitment of persons to the stakeholder group itself is set by the PSP lead and therefore can be beset by individual bias on who is recruited and from what backgrounds. Hence, there is the potential for significant cultural and social bias within the PSP even before the questionnaire stage [36,37]. Ultimately, increasing diversity within these groups will bring about cognitive diversity, which will improve the quality of the work.

**Figure 2:**
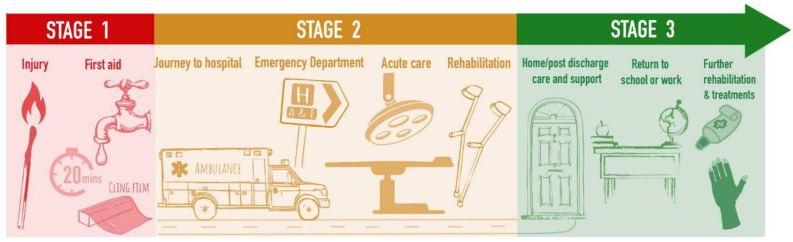
The burn care journey.

The PSP process focuses on clinical questions that ultimately impact patient care (Figure 2). However, this potentially ignores translational research that may revolutionise patient care. Furthermore, one limitation of this process is that it (for obvious reasons) focuses predominately on producing potential questions for clinical trials. However, this potentially ignores the huge amount of translational research that is required in medical fields, such as scarring. As such, it therefore becomes difficult for translational researchers to utilise these questions as evidence for the necessity of their fields or grant proposals, hence undermining the crucial bedside raison d’être that funders look for when approving any proposal.

Finally, research is a dynamic field. PSPs produce lists of static questions that perhaps should be updated as questions are engaged and answered. This would enable the field to focus on new questions much faster and to be more responsive to the dynamic needs of the population. As such, such priority lists are only revisited when self-nominated clinicians approach the task. Therefore, there is perhaps a lack of continuity in the ongoing stewardship of PSPs.

Despite this, the benefits of research prioritisation far outweigh the aforementioned drawbacks. Such exercises meet the aims of the WHO constitution and address the 10/90 gap in research needs and investments [38,39].

## 5. How Does Research Prioritisation Affect Scarring?

One of the research priorities likely to be ranked most highly, particularly by patients, is the prevention and management of burn-related scarring. Cutaneous scarring remains, for many patients, a long-term reminder of their burn injury with psychological, functional and aesthetic consequences for daily living [31,39,40,41]. Globally, 100 million patients acquire scars each year, of which approximately four million are due to burn injuries and 70% are in children [42,43]. In 2020, the global scar treatment market size was estimated at 12.3 billion [44]. Scarring is a multi-faceted disease that includes research on the pathophysiology, risk factors, psychology, assessment and management. Despite the high costs of scar management and the global healthcare burden of burn-related scarring, research in this area receives only a small fraction of the total healthcare research funding. In the UK, for example, health research funding of GBP 4.8 billion was invested in 2018 [45]. The largest funding increase was for infectious diseases. Cancer research received a substantial proportion of the total funding. Research into skin injuries and accidents together received only 1.2% of the total budget for UK healthcare research [45]. Topics ranked as priorities for research funding in cutaneous scarring might include standardisation and consensus on scar assessment, surgical and non-surgical approaches to improving function and cosmesis, and the management of associated psychological well-being [46].

A search of the EU clinical trials register reveals 68 results pertaining to scarring, which include trials on therapeutic interventions from pharmaceutical companies on hypertrophic scarring, keloids and hidradenitis suppurativa amongst other conditions. Alternatively, the database reveals 85 trials related to burns alone, focussing on dressings and analgesia.

There is no doubt that scarring is of critical importance to future research within burn care. The most pressing issue is deciding which clinical questions facing patients are most important, what could be answered through multi-centre trials and what is the key question underpinning the patient problem. Answering this prioritisation process does not preclude bench-side research into scarring or undermine its funding but provides the much-needed clinical output for the research pipeline in burn care. Scarring as a pathology is not only associated with burns but also associated with a huge variety of clinical conditions. One larger question is whether scarring as a broad condition deserves its own research prioritisations. Priorities concerning scar prevention and treatment in burn care are important to consider in the context of other research in burns. However, this may not serve the wider scar research and patient community well. Instead, it may be more fruitful to consider the entire range of scarring pathologies, which include unifying questions about the features amongst them and to produce a research priority list for that field. These are the kinds of important questions that research prioritisation exercises force us to consider, answer and take action on.

## 6. Conclusions

Scarring produces a huge burden both in burn care and more broadly in medicine. The social and economic costs of scarring are far reaching amongst patients and their families long after their initial injury. There are many treatment uncertainties in scarring, particularly burn scarring, with associated variation in patient management and outcomes. It is essential to assess what research topics and evidence gaps should be prioritised according to the people using the evidence on a regular basis [25]. The USA Patient-Centered Outcomes Research Institute (PICORI), and the UK National Institute for Health Research (NIHR) support co-production of research with patients and clinicians [18]. One approach is to consider the research priorities of scarring in the context of burn injuries. There has been no systematic and inclusive conversations between clinicians, patients and carers about priorities for clinical research in burn care. The hope is that by prioritising research uncertainties and by achieving consensus on this between healthcare staff and patients, the future research agenda of international funding organisations will be set and research that is needed in burn care will be funded and undertaken.
